# Barriers and Facilitators to Conducting Oncology Clinical Trials in the UAE

**DOI:** 10.3390/clinpract12060093

**Published:** 2022-11-07

**Authors:** Humaid O. Al-Shamsi

**Affiliations:** 1 Department of Oncology, Burjeel Cancer Institute, Burjeel Medical City, Abu Dhabi P.O. Box 92510, United Arab Emirates; alshamsi@burjeel.com or humaid.al-shamsi@medportal.ca; Tel.: +971-50-631-5388; 2 Innovation and Research Center, Burjeel Cancer Institute, Burjeel Medical City, Abu Dhabi P.O. Box 92510, United Arab Emirates; 3 College of Medicine, University of Sharjah, Sharjah P.O. Box 27272, United Arab Emirates; 4 Emirates Oncology Society, Dubai P.O. Box 6600, United Arab Emirates

**Keywords:** cancer, oncology, research, clinical trials, research and development, UAE

## Abstract

Cancer research and clinical trials are essential to improve cancer patients’ outcomes and advance the oncology field. The United Arab Emirates (UAE) has been lagging in cancer research with many barriers, including healthcare, institutional, regulatory, patient and community, the global oncology community, and the pharmaceutical industry. In this report, we try to address these challenges from our perspective. Making clinical trials accessible for cancer patients in the UAE requires a collaborative approach from all stakeholders and serious consideration for the greater cause to improve the patient’s outcome and contribute to the advancement of the cancer field worldwide. There has been significant support from the UAE government and the regulators in the UAE to facilitate and encourage research in general and cancer research in particular with recent initiatives and international collaborations. Private and public institutions must overcome their competitive moods and work together to strengthen the research network across the UAE and improve accrual for potential clinical trials. Public awareness and education must overcome long-standing perceptions about research and clinical trials in the UAE. The pharmaceutical industry must work closely with institutions across the UAE and support them in establishing accredited research programs and clinical trial units. The Emirates Oncology Society is establishing the Oncology Research Working Group to advocate and advance cancer research in the UAE. All stakeholders must be engaged to successfully implement impactful clinical trials in the UAE and the region.

## 1. Background

Between 1 January–31 December 2019, the total number of newly diagnosed cancer cases (malignant and in situ) reported to the United Arab Emirates National Cancer Registry (UAE-NCR) was 4633, of which 4381 (94.56%) were malignant and 252 (5.44%) were in situ cases. Overall, cancer was more prevalent among women than men; it affected 2604 (56.2%) females and 2029 (43.8%) males. Among UAE citizens, a total of 1193 cases were newly diagnosed with cancer, out of which 1117 (93.6%) were malignant and 76 (6.4%) were in situ cases. Similarly, in non-UAE citizens, 3440 cases were newly diagnosed with cancer, of which 3264 (94.9%) were malignant, and 176 (5.1%) were in situ cases, representing an overall crude incidence rate of 46.1/100,000 for both genders. There is a clear female predominance in cancer incidence. The crude incidence rate was higher for females (75.8/100,000) than for males (31.0/100,000). The overall age-standardized incidence rate (ASR) was 78.4/100,000. Breast, thyroid, colorectal, skin, and leukemia were the top-ranked cancers among all new cancer cases in both genders. Colorectal, skin, prostate, leukemia, and non-Hodgkin’s lymphoma were the top-ranked cancers among males. Among females, breast, thyroid, colorectal, uterus, and ovary were the top-ranked cancers. In the year 2019, there were 125 children in the age group of 0–14 years diagnosed with new cancer in the UAE (54% were females and 46% were males). This constitutes about 2.9% of all registered malignant cases. Leukemia, brain and CNS, connective and soft tissue, non-Hodgkin’s lymphoma, and bone and articular were the most common cancers in boys and girls. The third leading cause of death in the UAE, after diseases of the circulatory system and injuries, was found to be cancer. The number of deaths from cancer totaled 1181 (629 in males and 552 in females) and accounted for 13.11% of all deaths regardless of nationality, type of cancer, or gender. This represents an estimated age-standardized mortality rate of 33.3 deaths per 100,000 for both genders. Breast cancer was the leading cause of cancer death in 2019, with an estimated average of 11.6% of cancer deaths per year; colon cancer was the second most common cause of cancer death in both sexes, and lung cancer was the third most common cause of cancer death in both sexes [1].

Cancer care in the UAE has considerably evolved over the last two decades. However, advances in cancer research have notably lagged behind [2]. The USA and Europe are leading the cancer research world with significant research and development (R&D) programs; these programs have been long-established, well structured, and have received global recognition for their contribution to cancer research and improving patient outcomes. Oncology trials worldwide reached historically high levels in 2021, up 56% from 2016, and mostly focused on rare cancer indications [3,4]. No previous publications have addressed the barriers and facilitators to conducting cancer research in general and clinical trials in the United Arab Emirates (UAE). For perspective and to take breast cancer as an example, we conducted a dedicated and systematic literature search to identify publications related to breast cancer from the UAE. We searched PubMed using the terms “breast” AND “Cancer* OR Oncol* OR malignant* OR tumor OR tumor” AND “emirates OR UAE” on 8 August 2022. A total of 203 journal publications by authors from the UAE were retrieved, with the earliest publication being from 2001. The majority were basic science/translational (45.8%) or observational (26.1%) studies, while 40 (19.1%) were non-data-driven publications (e.g., reviews, consensus statements, editorials), and only five clinical trials were identified. Of note, among the 163 data-driven publications, only 62 (38%) were performed in the UAE, while the remaining were conducted abroad with authors having a UAE affiliation. Only seven clinical trials were identified by expanding the search to all cancers (Table 1).

We conducted a further search on clinicaltrials.gov to identify clinical trials that may not have been published; this search was done on 21 October 2022, and we identified 25 studies. There were 15 completed trials, 6 actively recruiting, and 3 of unknown status (Table 2).

**Figure 1:**
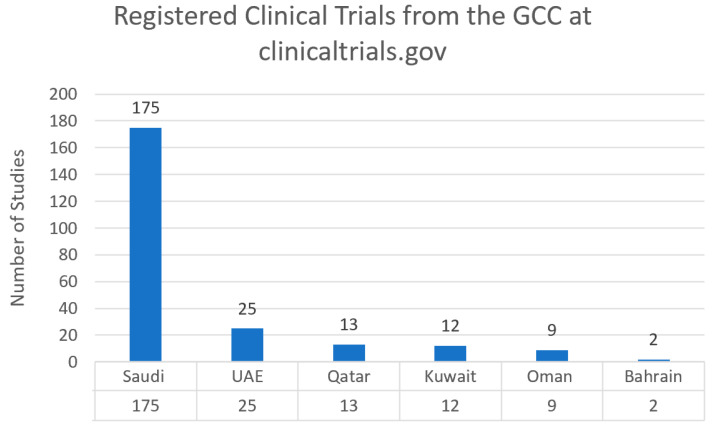
Registered clinical trials from the GCC countries at clinicaltrials.gov as of 21 October 2022.

Cancer research activities varied between the Gulf Cooperation Council Countries (UAE, Saudi Arabia, Oman, Bahrain, Qatar, and Kuwait). We conducted a search for oncology clinical trials in each country using clinicaltrials.gov. Saudi Arabia had the highest number of registered clinical trials with 175 trials, followed by the UAE with 25 registered clinical trials (Figure 1).

We herein provide perspectives on some of the reasons for such limited participation and conduct of clinical trials in the UAE while highlighting some unique opportunities.

## 2. Healthcare Provider Barriers

**Figure 2:**
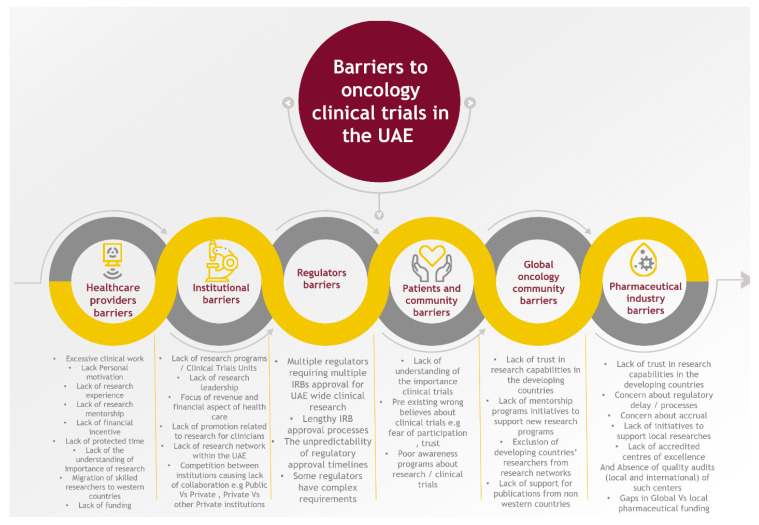
Barriers to oncology clinical trials in the UAE.

Healthcare providers are the cornerstone for clinical research; they identify the clinical question in their patient population that needs to be addressed in clinical trials and are responsible for the accrual of patients, the clinical assessment of treatment toxicity, and eventually the implementation of the clinical trial results in their patients. Many studies have assessed barriers to clinical research from healthcare providers’ perspectives. The following factors were identified: excessive clinical work, lack of personal motivation, lack of research experience, lack of research mentorship, lack of financial incentive, lack of protected time, and lack of understanding of the importance of research [12,13,14,15] (Figure 2). Most of these barriers related to healthcare providers in the UAE were also shared and highlighted in a survey to clarify UAE nurses’ perceptions of barriers to implementing research in clinical practice in the UAE. The two highest-ranked barriers to nurses conducting research in the UAE were a lack of time and competing demands for time [16] (Figure 2). Due to the lack of clear clinical oncology research programs in the UAE, skilled researchers and scholars are potentially migrating to Western countries for better opportunities; with more research programs, more opportunities will attract and retain researchers in the UAE and the wider region. Lastly, the medical school curriculum, residency, and fellowship training must include specific training and education for medical students and health care trainees to obtain research skills and experience.

## 3. Institutional Barriers

Institutions play a critical role in the ecosystem of oncology research and clinical trials. The lack of research programs and clinical trials units (CTUs) in health care institutions contributes immensely to the progress of cancer research in the UAE. Only a few cancer centers (5 out of 33) in the UAE have ongoing research and clinical trials. Establishing and activating such units is a limiting factor for cancer research. Lack of research leadership is another limitation. With the focus of institutions on meeting financial targets, less attention is given to clinical research. Furthermore, the competition between institutions may lead to poor collaboration between private institutions or between private and public institutions. To overcome this, institutions should see the value and potential of research networking and collaboration within the UAE (Figure 2). Lastly, the lack of promotion related to research productivity for clinicians must be evaluated and readdressed to increase the clinician’s interest and involvement.

## 4. Regulatory Barriers

In the UAE, multiple regulatory agencies exist (for the whole country and by Emirate), which means several approvals may need to be sought to conduct clinical research. In some instances, approvals may be lengthy or come with unpredictable approval timelines, although this is changing with the expansion of medical research teams in regulatory agencies and with optimized and more efficient procedures. The Dubai Health Authority, through the Dubai Scientific Research Ethics Committee (DSREC), follows the following general guidelines for research approvals in Dubai: Phase 1—not approved to be conducted in DHA; Phase 2—requires review and approval by higher management of the Dubai Health Authority; Phase 3 and Phase 4—DSREC review and a favorable opinion for the study submitted [17]. The Department of Health (DOH) in Abu Dhabi does allow the conduct of phases I and II trials in addition to phases III and IV [18,19]. The DOH has been leading research efforts at the regulatory level to enhance and improve the research capabilities in Abu Dhabi [20,21]. The Statistics and Research Center at the Ministry of Health and Prevention allows all types of clinical research with a more in-depth review before approval for phase I and II trials [22].

## 5. Patients and Community Barriers

With the lack of research and clinical trial culture in the medical community in the UAE, a lack of understanding of the importance of clinical trials is the norm among patients and the community. This leads to lower acceptance rates for enrollment in clinical trials, even if trials are accessible in the UAE. This is usually coupled with pre-existing wrong beliefs about clinical trials, e.g., fear of experimentation and trust issues. To change this, a strong awareness program about research/clinical trials is necessary (Figure 2).

## 6. Global Oncology Community Barriers

The global oncology community has an important role in supporting knowledge transfer and the involvement of researchers from the UAE and the region in designing and conducting clinical trials. However, the lack of trust in research capabilities in developing countries and the exclusion of researchers from those countries from research networks is another significant barrier. The lack of support for publications from developing countries in reputable journals is also a primary challenge [23]. The global community should continue to support and create mentorship programs and initiatives to support new research programs in the UAE and other countries (Figure 2).

## 7. Pharmaceutical Industry Barriers

Pharmaceutical research and development funding play a significant role in advancing cancer research and drug discovery [24]. The pharmaceutical industry’s support for research in some regions and countries, including the UAE, faces many challenges and barriers. This mainly stems from a lack of trust in research capabilities, concern about regulatory delays/processes, a limited/lack of accredited centers of excellence, and the absence of quality audits (local and international) of such centers. There is also a concern about successful accrual if clinical trials open in the UAE. The above challenges led to a lack of initiatives to support local research [18] and gaps in global vs. local pharmaceutical funding for research and investigator-initiated trials [25].

## 8. Conclusions

We have summarized barriers and potential facilitators for conducting oncology trials in the UAE, which may apply to other countries in the Middle East and Asia. There is a need for collaborative efforts from all stakeholders to address all these challenges. Efforts in cancer research are developing, although numerous evidence gaps remain. With the continuous growth in academic institutions and research programs committed to cellular and molecular research initiatives, the UAE’s scope for basic and translational research is typically improving. However, clinical research in general and clinical trials specifically are far from ideal in quantity and focus. There is an urgent need for a call to action from the local and global communities to optimize the inclusion of the UAE in international clinical trials and to provide local funding opportunities for local studies so that the efficacy and safety of drugs are evaluated in the local population rather than “assumed” from data from Western countries. The Emirates Oncology Society is establishing an oncology research group to advocate and advance cancer research in the UAE. For the success of this initiative, all stakeholders must support each other and work together for better cancer care in the UAE.

## Figures and Tables

**Table 1:** Clinical trials in cancer indications conducted in the UAE. RCT, randomized, controlled trial.

Reference	Year	Cancer	Phase	Number of Subjects	Arab Countries Participated
[5]	2003	Hematological malignancy	III	40	UAE only
[6]	2006	Hematological malignancy	II	20	UAE only
[7]	2016	Breast	II	80	Saudi Arabia, Kuwait, Egypt, UAE
[8]	2017	Breast	III	2577	Algeria, Egypt, Morocco, Saudi Arabia, UAE
[9]	2018	Breast	III	672	Lebanon, Saudi Arabia, UAE
[10]	2021	Breast	III	1436	Algeria, Egypt, Lebanon, Morocco, Saudi Arabia, UAE
[11]	2021	Breast	III	258	Algeria, Egypt, Jordan, Lebanon, Morocco, Saudi Arabia, Tunisia, UAE

**Table 2:** Clinical trials in the UAE registered at clinicaltrials.gov as of October 2022.

Title	Identifier	Condition	Location	Status	Phase	Publication Indexed to Relevant NCT Number
Pembrolizumab And Tamoxifen Among Women With Advanced Hormone Receptor Positive Breast Cancer And Esr1 Mutation (Pembro)	NCT03879174	Breast cancer—female	Mediclinic City Hospital, UAE	Unknown	2	22149876, 27959613, 24185512, 20479064
Prevalence of BRCA1 and BRCA2 Mutations in Ovarian Cancer Patients in the Gulf Region (PREDICT)	NCT03082976	Ovarian cancer	Al Ain, UAE (PI is from Tawam)	Completed	N/A	34930165
Epidemiological Study to Describe Non Small Cell Lung Cancer Clinical Management Patterns in MENA. Lung-EPICLIN/ Gulf (Lung-EPICLIN/G)	NCT01562665	Non-small cell lung cancer	Al-Ain, Abu Dhabi (UAE) PIs are from Zayed Military Hospital and Tawam Hospital)	Completed	N/A	N/A
Retrospective Study to Describe the Real-world Treatment Patterns and Associated Clinical Outcomes in Patients With Metastatic Castration-resistant Prostates Cancer (REMPRO)	NCT04801186	Metastatic castration-resistant prostate cancer	Dubai and Abu Dhabi, UAE	Recruiting	N/A	N/A
Retrospective Epidemiology Study Of ALK Rearrangement In Non-Small Cell Lung Cancer Patients In The Middle East & North Africa (ALK NSCLC MENA)	NCT02304406	Non-small cell lung cancer	Tawam Hospital, Al-Ain/Al-Maqam (UAE)	Completed	N/A	N/A
Phase III Trial of High Dose vs. Standard Dose Vit D2 With Docetaxel in Met Breast ca (GORG-002)	NCT00944424	Breast cancer	Tawam Hospital, Al Ain, UAE	Unknown	3	N/A
Study to Determine the Prevalence of Homologous Recombination Deficiency Among Women With Newly Diagnosed, High-grade, Serous or Endometrioid Ovarian, Primary Peritoneal, and/or Fallopian Tube Cancer (HALO)	NCT04991051	Fallopian tube cancer	Al-Ain, UAE	Completed	N/A	N/A
Fulvestrant Versus Fulvestrant Plus Palbociclib in Operable Breast Cancer Responding to Fulvestrant (SAFIA)	NCT03447132	Breast neoplasm—female	Tawam Hospital, Al-Ain, UAE	Completed	3	N/A
A Study of Trastuzumab Emtansine in Participants With Human Epidermal Growth Factor Receptor 2 (HER2)-Positive Breast Cancer Who Have Received Prior Anti-HER2 And Chemotherapy-based Treatment	NCT01702571	Breast cancer	UAE	Completed	3	34741021, 32634611
Study of Efficacy and Safety in Premenopausal Women With Hormone Receptor Positive, HER2-negative Advanced Breast Cancer (MONALEESA-7)	NCT02278120	Advanced metastatic breast cancer	Novartis Investigative Site, Al Ain, UAE	Active, not recruiting	3	34965945, 34504990, 34158598, 33769862, 31305131, 31166679, 29804902
A Study of Pertuzumab in Combination With Trastuzumab (Herceptin) and a Taxane in First-Line Treatment in Participants With Human Epidermal Growth Factor 2 (HER2)-Positive Advanced Breast Cancer (PERUSE)	NCT01572038	Breast neoplasms	Tawam Hospital, Al-Ain, UAE	Completed	3	30796821
A Safety and Tolerability Study of Assisted and Self-Administered Subcutaneous (SC) Herceptin (Trastuzumab) as Adjuvant Therapy in Early Human Epidermal Growth Factor Receptor 2 (HER2)-Positive Breast Cancer (SafeHER)	NCT01566721	Breast neoplasms	Tawam Hospital, Al-Ain, UAE	Completed	3	30018134, 28625777
Chemotherapy Plus Surgery in Treating Children at Risk of or With Stage I Wilms’ Tumor	NCT00003804	Kidney cancer	Tawam Hospital, UAE	Unknown	3	10463285, 19300220, 17084075, 15542800, 15175957, 20582946, 18811224, 22238153, 22513793, 21509706, 21370404, 21670612, 18095319, 17162067, 16410128
A Study of Bevacizumab in Combination With Standard of Care Treatment in Participants With Advanced Non-squamous Non-small Cell Lung Cancer (NSCLC)	NCT01351415	Non-squamous, non-small cell lung cancer	Tawam Hospital, Al-Ain, UAE	Completed	3	30177994, 26828788, 21705281
AVAPERL1 Study: A Study of Avastin (Bevacizumab) With or Without Pemetrexed as Maintenance Therapy After Avastin in First Line in Patients With Non-Squamous Non-Small Cell Lung Cancer	NCT00961415	Non-squamous, non-small cell lung cancer	Al-Ain, UAE	Completed	3	23835708
Liposomal Amphotericin B in Treating Granulocytopenia and Persistent Unexplained Fever in Cancer Patients	NCT00003938	Cancer	Tawam Hospital, UAE	Completed	3	N/A
Prevention of Colorectal Cancer Through Multiomics Blood Testing (PREEMPT CRC)	NCT04369053	Colon cancer, rectal cancer, colon neoplasm, colon diseases, colon lesion, colon polyp, colorectal cancer, polyp, adenoma, rectal diseases, gastrointestinal tract cancers	Cleveland Clinic Abu Dhabi, Abu Dhabi, UAE	Recruiting	N/A	N/A
A Study Investigating the Outcomes and Safety of Atezolizumab Under Real-World Conditions in Patients Treated in Routine Clinical Practice (IMreal)	NCT03782207	Urothelial carcinoma, non-small cell lung cancer, small cell lung cancer, hepatocellular carcinoma	NMC Speciality Hospital, Abu Dhabi, UAE Tawam Hospital, Al-Ain, UAE	Recruiting	N/A	N/A
A Study of Atezolizumab (Tecentriq) to Investigate Long-term Safety and Efficacy in Previously-treated Participants With Locally Advanced or Metastatic Non-small Cell Lung Cancer (NSCLC) (TAIL)	NCT03285763	Carcinoma, non-small-cell lung cancer	Tawam Hospital, Al-Ain, UAE	Completed	4	33737339
Phase III Study of Lenalidomide and Dexamethasone With or Without Elotuzumab to Treat Relapsed or Refractory Multiple Myeloma (ELOQUENT-2)	NCT01239797	Lymphoma, multiple myeloma	Local Institution, Leicester, United Arab Emirates, LE1 5WW	Completed	3	30204239, 28070715, 26035255
Prospective Urban Rural Epidemiology Study (PURE)	NCT03225586	Cardiovascular diseases, risk factor, cardiovascular, health behavior, environmental exposure, lung diseases, cancer, injuries, renal disease, communicable disease	Dubai Medical University, Dubai, UAE	Recruiting	N/A	36088949, 34261638, 32546275, 31965140, 30149823, 29734966
Safety and Efficacy of Nilotinib in Newly Diagnosed Chronic Myeloid Leukemia Patients (ENESTxtnd)	NCT01254188	Chronic myeloid leukemia	Novartis Investigative Site, Dubai, UAE	Completed	3	28699641
An Observational Study to Evaluate the Real-World Clinical Management and Outcomes of ALK-Positive Advanced NSCLC Participants Treated With Alectinib (ReAlec)	NCT04764188	NSCLC	Mediclinic Airport Road Hospital, Dubai, UAE Mediclinic City Hospital, Dubai, UAE	Recruiting	N/A	N/A
Global Investigation of Therapeutic Decisions in Hepatocellular Carcinoma and of Its Treatment With Sorafenib (GIDEON)	NCT00812175	Carcinoma, hepatocellular	Many locations, UAE	Completed	N/A	29499662, 26901163, 20642705
Multicentre Registry of Treatments and Outcomes in Patients With Chronic Lymphocytic Leukaemia (CLL) Or Indolent Non Hodgkin’s Lymphoma (iNHL) (NADIR)	NCT02273856	Indolent non-Hodgkin’s lymphoma (iNHL), chronic lymphocytic leukemia (CLL)	Site AE97101 Sheikh Khalifa Medical City, Abu Dhabi, United Arab Emirates, 51900	Terminated (The study was terminated due to insufficient subject enrollment and very slow enrollment.)	N/A	N/A

## Data Availability

Not applicable.
